# Tubules of plant reoviruses exploit tropomodulin to regulate actin-based tubule motility in insect vector

**DOI:** 10.1038/srep38563

**Published:** 2017-01-09

**Authors:** Qian Chen, Linghua Zhang, Yanshuang Zhang, Qianzhuo Mao, Taiyun Wei

**Affiliations:** 1 Fujian Province Key Laboratory of Plant Virology, Institute of Plant Virology, Fujian Agriculture and Forestry University, Fuzhou, Fujian 350002, P.R. China

## Abstract

Plant reoviruses are known to exploit virion-packaging tubules formed by virus-encoding non-structural proteins for viral spread in insect vectors. Tubules are propelled by actin-based tubule motility (ABTM) to overcome membrane or tissue barriers in insect vectors. To further understand which insect factors mediate ABTM, we utilized yeast two-hybrid and bimolecular fluorescence complementation assays to test interactions between tubule protein Pns10 of rice dwarf virus (RDV), a plant reovirus, and proteins of its insect vector, the leafhopper *Nephotettix cincticeps*. Tropomodulin (Tmod), vitellogenin, and lipophorin precursor of *N. cincticep* displayed positive and strong interaction with Pns10, and actin-associated protein Tmod interacted with Pns10 in pull-down assay and the co-immunoprecipitation system. Further, we determined Pns10 tubules associated with Tmod in cultured cells and midgut of *N. cincticep*. The expression dynamic of Tmod was consistent with that of Pns10 and the fluctuation of RDV accumulation. Knockdown of Tmod inhibited the Pns10 expression and viral accumulation, thus decreasing the viruliferous rates of leafhopper. These results suggested that Tmod was involved in viral spread by directly interacting with Pns10 tubules, finally promoting RDV infection. This study provided direct evidence of plant reoviruses utilizing an actin-associated protein to manipulate ABTM in insect vectors, thus facilitating viral spread.

Plant reoviruses encode a non-structural protein which assembles into tubules by self-interaction, for example, southern rice black-streaked dwarf virus (SRBSDV) from the genus *Fijivirus* encodes the protein P7-1, rice dwarf virus (RDV) and rice gall dwarf virus (RGDV) of the genus *Phytoreovirus* encodes Pns10 and Pns11, respectively^1^,^2^,^3^,^4^. These tubules approximately 85 nm in diameter, regularly fix the viral particles on the inner surface, and exploit the actin-based tubule motility (ABTM) to positively facilitate viral spread in insect vectors^4^,^5^. Studies on infected cultured cells of insect vectors show that the virions-packaging tubules, which are propelled by the power from the ABTM, insert the actin-based filopodia and extend into the neighboring insect cells for viral cell-to-cell dissemination^1^,^2^,^3^. In alimentary canal of the insect vectors, the growing tubules take advantage of ABTM to pass through actin-based microvilli of the epithelium into the lumen, or cross the basal lamina from the initially infected epithelium, or traverse the tight junctions between epithelial cells for facilitating viral intercellular spread^1^,^4^,^6^. The tubules further move along the actin-based visceral muscles of the whole intestine for viral lateral spread in the aid of ABTM^1^,^4^,^6^. Therefore, by means of ABTM, tubules serve as a powerful and efficient tool for the spread of plant reoviruses by overcoming membrane or tissue barriers within insect vectors. However, the underlying mechanism involved in the ABTM, the interaction between the virus-containing tubules and the actin-based cellular machinery of the insect vectors for efficient transmission of the virus still remains unclear.

RDV, a member of the *Phytoreovirus* genus in the family *Reoviridae*, contains 12 double-stranded RNA (dsRNA) segments (S1-S12) encoding seven structural proteins (P1, P2, P3, P5, P7, P8 and P9) and five nonstructural proteins (Pns4, Pns6, Pns10, Pns11 and Pns12)^7^,^8^. RDV is mainly transmitted by the leafhopper vector, *Nephotettix cincticeps*, in a persistent-propagative manner^9^. In *N. cincticeps*, the infection route of RDV has been revealed as follows: RDV virions are ingested and access to the alimentary canal via the stylet and esophagus^10^. The viral particles specifically recognize and bind to the receptors on the epithelium of filter chamber, and subsequently enter the epithelial cells via endocytosis^11^. Replication of the virus is initiated after the nonstructural proteins Pns6, Pns11 and Pns12 aggregate together to form the viroplasm matrix^12^. The Pns12 protein serves as a principal regulator for viral replication and infection in its insect vector, while Pns4, a phosphoprotein forms minitubules and localizes around the viroplasm matrix, essential for viral infection and replication in insect vector^12^,^13^,^14^,^15^. Pns10 proteins assemble to form a tubule into which the progeny virions maturing at the periphery of viroplasm are packaged. In the initially infected epithelial cells of the filter chamber, virus-containing Pns10 tubules pass through actin-based microvilli into the lumen and extend to neighboring cells^2^,^6^,^16^. After passing through the basal lamina, Pns10 tubules move along the actin-based circular muscles and longitudinal muscles for lateral spread of RDV throughout the gut^6^. Therefore, RDV utilizes Pns10 tubules to overcome multiple barriers in the infection route from intestine to salivary glands of *N. cincticeps*^6^,^10^. Moreover, the RDV isolate deficient in Pns10 protein expression fails to be transmitted by the leafhopper vector^17^, suggesting Pns10 to be responsible for viral spread and transmission by the insect vector. Recent studies suggest that the interaction between Pns10 and cytoplasmic actin of efficient vector *N. cincticeps* is correlated with insect vector specificity^18^, suggesting that Pns10 tubule is capable of employing components from its vector to overcome membrane or tissue barriers for viral transmission. Nevertheless, the mechanism of Pns10 tubule exploiting the actin-based cellular machinery in the insect body to spread and transmit virions is still unknown.

This study investigated the molecular mechanisms involved in the spread of RDV Pns10 tubules, by applying various molecular techniques such as yeast two-hybrid assay (YTH), bimolecular fluorescence complementation (BiFC), pull-down assay, the co-immunoprecipitation (Co-IP) and RNA interference (RNAi). Putative interactors from the leafhopper vector with the viral non-structural protein Pns10 were screened, and 6 putative proteins have been examined for their interaction with Pns10 in different interaction systems. An actin-associated protein was identified to strongly interact with Pns10, and served as a positive regulator for the spread of Pns10 tubules.

## Results

### Identification of putative interactors from *N. cincticeps*

A YTH assay was performed to screen for putative candidates interacting with RDV Pns10. A cDNA library was constructed using the adults of *N. cincticeps* as the source of mRNA, and the titer of the primary cDNA library was 4.0 × 10^6^ cfu/17 μL. The average insert size was 1.5 kb, meeting the requirement of a standard cDNA library.

The gene of RDV Pns10 was cloned into the pGBKT7 to generate the bait pGBKT7-Pns10. Mating yeast containing bait pGBKT7-Pns10 and positive prey plasmid resulted in reporter gene activation, then caused the colonies turn blue on Quadruple dropout media (QDO) plates containing X-α-Gal. Finally, 232 positive colonies were picked from this library screen, and 149 colonies were randomly sequenced. Of these, 47 sequences were annotated using the BLASTX searching method in the GenBank (Supplementary Table S1). The species distribution associated with best match for each sequence annotated was depicted in Fig. S1a. The screening frequency of the sequences displayed a high representation for putative vitellogenin (Vg), followed by enzymes, transcription factors and ribosomal RNA genes (Supplementary Fig. S1b). Gene Ontology (GO) annotation analysis demonstrated that these proteins were classified into 16 molecular function groups: 25% with transporter activity, 12.5% with protein binding or translation regulator activity, 9.4% with nucleic acid binding activity, 6.3% with transcription factor activity, and 3.1% with endonuclease activity or others (Supplementary Fig. S2). The function of putative candidates with the highest percentage was transporter activity, which was consistent with the role of Pns10 tubules in the spread of virions.

### Pns10 interacts with putative candidate vector proteins *in vitro*

**Figure 1:**
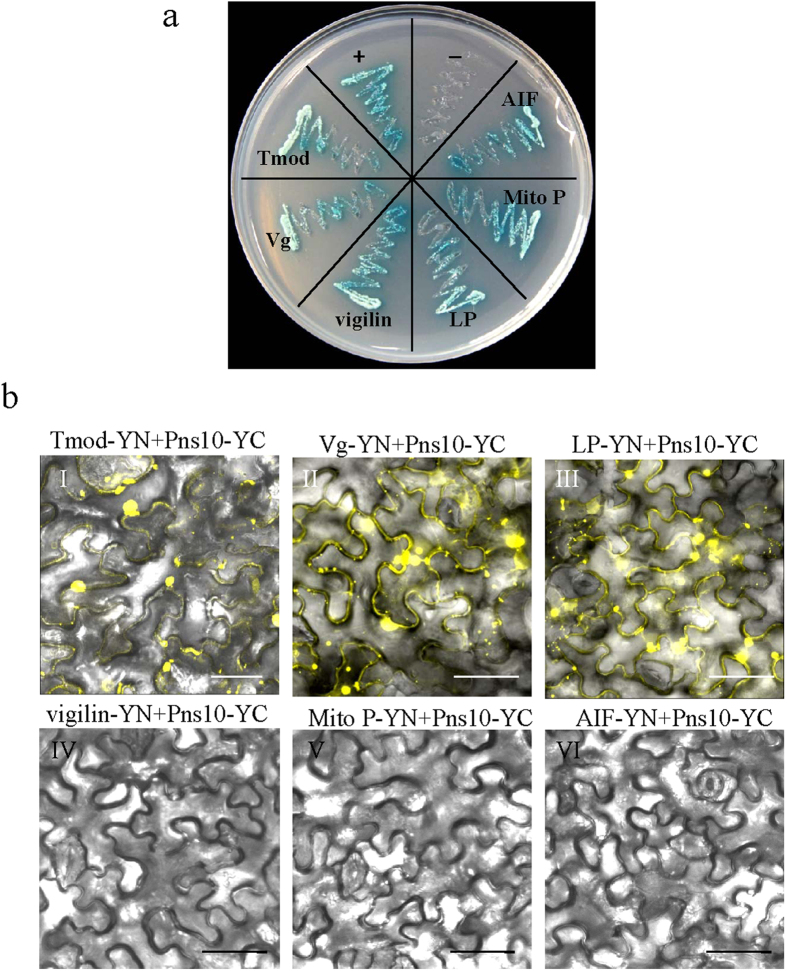
Interaction of Pns10 with candidate proteins in YTH and BiFC assays. (**a**) YTH assay of Pns10-candidate protein interactions. Transformants on plate of SD-Trp-Leu-His-Ade medium were as follows. +, Positive control, i.e., pGBKT7-53/ pGADT7-T; –, negative control, i.e., pGBKT7-Lam/pGADT7-T; Tmod, pGBKT7-Pns10/pGADT7-Tmod; Vg, pGBKT7-Pns10/pGADT7-Vg; LP, pGBKT7-Pns10/pGADT7-LP; vigilin, pGBKT7-Pns10/pGADT7-vigilin; Mito P, pGBKT7-Pns10/pGADT7-Mito P; AIF, pGBKT7-Pns10/pGADT7-AIF. (**b**) BiFC assays of Pns10-candidate protein interactions in leaf cells co-expressing Tmod-YN and Pns10-YC (I), Vg-YN and Pns10-YC (II), LP-YN and Pns10-YC (III), vigilin-YN+Pns10-YC (IV), Mito P-YN+Pns10-YC (V), and AIF-YN+Pns10-YC (VI). Images with yellow fluorescence were merged under a background of transmitted light. Bars, 40 μm.

Based on molecular functions and high screening frequency, 6 putative candidates which possibly played roles in viral spread in leafhopper vector were taken for further analysis. These included tropomodulin (Tmod), Vg, vigilin, lipophorin precursor (LP), mitochondrial porin (Mito P), and apoptosis-inducing factor (AIF). Six prey plasmids were identified in the YTH system again, and showed strong interaction with RDV Pns10 (Fig. 1a).

To validate the interactions of Pns10 with these 6 putative candidates, BiFC assays were carried out by co-expressing Pns10-YC and Tmod-YN, Pns10-YC and Vg-YN, Pns10-YC and LP-YN, Pns10-YC and vigilin-YN, Pns10-YC and Mito P-YN, or Pns10-YC and AIF-YN in *Nicotiana benthamiana* leaf cells. The strong BiFC signals were detected within the cytoplasm from the combinations of Pns10 and Tmod, Pns10 and Vg, or Pns10 and LP (Fig. 1b). In contrast, vigilin-YN, Mito P-YN, or AIF-YN did not show clear BiFC signals after co-expression with Pns10-YC (Fig. 1b). Our results indicated that Tmod, Vg and LP of *N. cincticeps* could specifically interact with the tubule protein Pns10 of RDV in the YTH system and BiFC assays.

### Pns10 interacts with actin-associated protein Tmod

Among three putative interactors of Pns10 *in vitro*, Tmod is a capping protein which partly regulates the length and dynamics of actin-tropomyosin filament by binding to the pointed end^19^,^20^. Therefore, it was reasonable to assume that this actin-associated protein might contribute to regulating Pns10 tubules growth, and hence we took it for further study.

**Figure 2:**
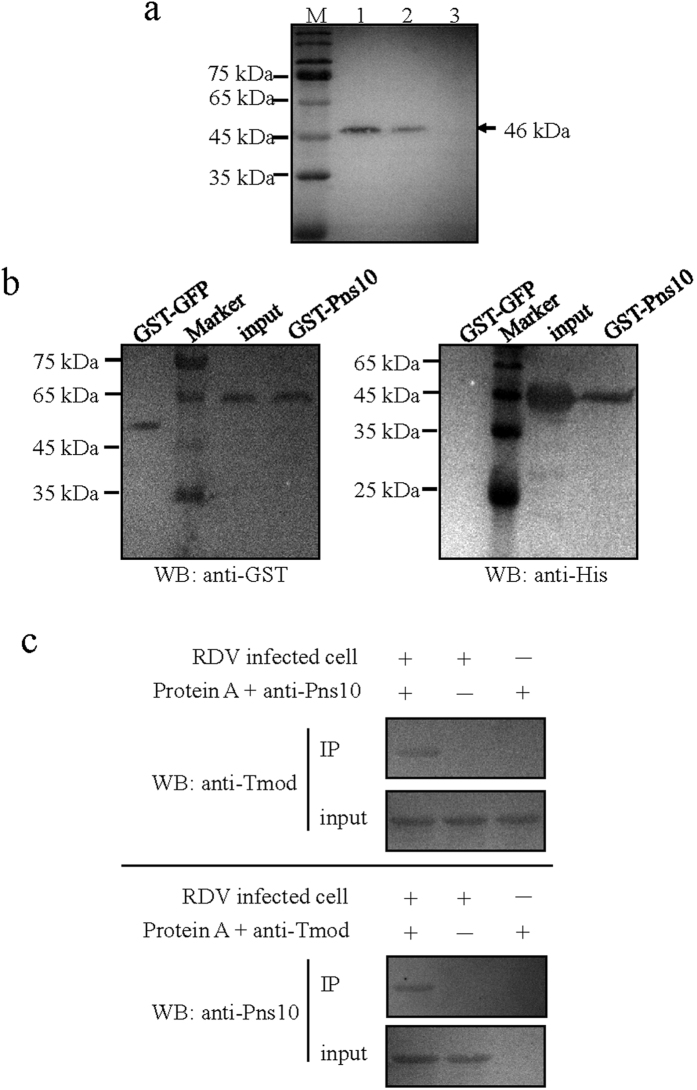
Pns10 specifically interacted with Tmod. (**a**) Western blotting analysis of Tmod antibodies specificity. Samples were separated by SDS-PAGE and detected with Tmod-specific antibodies. Lane M, protein marker; lanes 1 and 2, protein extracts from cultured cells and insect bodies; lane 3, negative control, i.e., PBS. (**b**) GST pull-down assay to detect interaction of Pns10 with Tmod. Recombinant protein GST-Pns10 or GST-GFP was incubated with cell lysate expressing the prey protein His-Tmod, respectively. Pull-down products were analyzed by Western blotting; the antibody against to GST was used to detect Pns10 and GFP, antibody against to His was used to detect bound proteins. (**c**) Co-IP assay to detect interaction of Pns10 with Tmod. The lysates of RDV-infected and mock-infected cultured cells were used for detection. Co-IP and Western blotting were performed using anti-His antibodies after the immune complex was precipitated on the resin to which anti-Pns10 was immobilized, using anti-Pns10 antibodies after the immune complex was precipitated on the resin to which anti-Tmod was immobilized. Full-length blots are presented in Supplementary Fig. S3.

The mouse polyclonal antibodies against the Tmod fused with His-tag were firstly prepared, and the specificity was tested using Western blotting assay. As expected, His-Tmod fusion protein was detected as a 46-kDa protein in extracts from virus-infected cultured cells and insect vectors, but not in the negative control (Fig. 2a), confirming the specificity of antibodies against the Tmod of *N. cincticeps*.

We subsequently performed GST pull-down assays and Co-IP experiments to understand whether Pns10 indeed interacted with Tmod. The results of GST pull-down assays were presented in (Fig. 2b), and indicated that the purified GST-Pns10 protein had the ability to pull down His-Tmod from cell lysates *in vitro*, while no such interaction was observed with the purified GST-green fluorescence protein (GFP). The results of Co-IP assays showed that when the anti-Pns10 antibodies were immobilized, the presence of Tmod was detected in immunoprecipitate by Western blotting using anti-Tmod antibodies as primary antibody and anti-mouse IgG antibodies as secondary antibody (Fig. 2c). In contrast, Tmod was not detected in the eluent of control resin without antibodies. We also verified the presence of Pns10 in immunoprecipitate by Western blotting using anti-Pns10 antibodies after immobilization of the anti-Tmod antibodies (Fig. 2c). To test interaction of Pns10 with Tmod was not caused by nonspecific binding, mock-infected cultured cells were utilized as negative control. Tmod was neither detected in the anti-Pns10 immunoprecipitates, nor was Pns10 detected in the anti-Tmod immunoprecipitates (Fig. 2c). Thus, we concluded the interaction of Pns10 with actin-associated protein Tmod.

### Co-localization of Tmod and Pns10 in *N. cincticeps*

**Figure 3:**
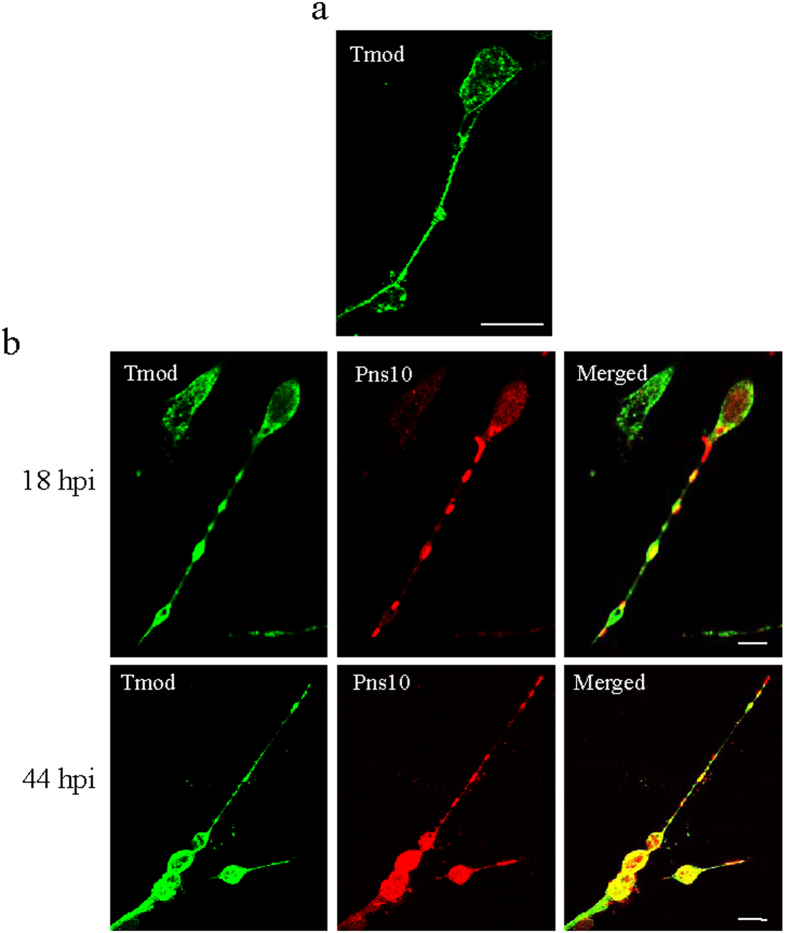
Co-localization of Tmod with Pns10 tubules of RDV in cultured cells of *N. cincticeps*. (**a**) Tmod localized with the filopodia of mock-infected cultured cells. (**b**) Tmod co-localized with Pns10 tubules in RDV-infected cultured cells. At 18 or 44 hpi, cells were immunolabeled, for Tmod with Tmod-FITC (green) and for Pns10 tubules with Pns10-rhodamine (red), then examined by confocal microscopy. Bars, 10 μm.

Then, immunofluorescence assays were performed to detect the expression and location of Tmod. At different hour post inoculation (hpi), infected cultured cells were treated with Tmod-specific IgG conjugated to fluorescein isothiocyanate (Tmod-FITC) and Pns10-specific IgG conjugated to rhodamine (Pns10-rhodamine), and then examined by immunofluorescence microscopy. In mock-infected cells, Tmod localized the filopodia extending toward neighboring cells (Fig. 3a), while in infected cells, Pns10 tubules protruded to adjacent cells along with the filopodia, as previously described^2^. At 18 hpi, Pns10 tubules co-localized with Tmod on the filopodia, to penetrate the cytoplasm of adjacent cells (Fig. 3b). At 44 hpi, with the accumulation of Pns10, more Tmod and Pns10 tubules co-localized on filopodia and cellular surface (Fig. 3b). Thus, Tmod associated with Pns10 tubules in cultured cells of insect vector.

**Figure 4:**
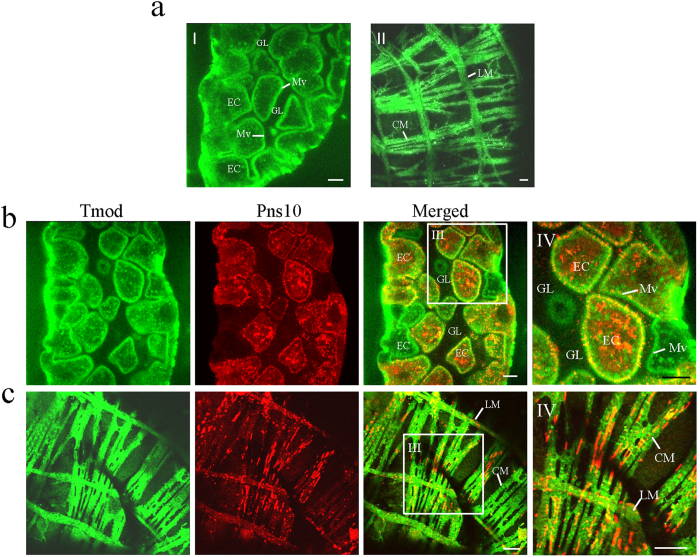
Tmod expressed and co-localized with Pns10 tubules of RDV in viruliferous insect vector. (**a**) Tmod localized to the microvilli of epithelial cells in the lumen side (I) and visceral muscles in the muscle side (II) of the gut in the nonviruliferous insects. In viruliferous insects, Tmod co-localized with Pns10 tubules at the microvilli of epithelial cells at 6-day (**b**) and visceral muscles 12-day padp (**c**) in the gut. Leafhopper organs were immunolabeled for Pns10 tubules with Pns10-rhodamine (red), and for Tmod with Tmod-FITC (green), then examined by confocal microscopy. Panel IV shows the enlarged images of boxed areas in panels III. EC, epithelial cell. Mv, microvilli. GL, gut lumen. CM, circular muscle. LM, longitudinal muscle. Bars, 10 μm.

We further examined the co-localization of Tmod and Pns10 in viruliferous leafhopper vector. In non-viruliferous insects, Tmod localized the microvilli along the surface of epithelia cells in the midgut and visceral muscles on the outer side of the midgut (Fig. 4a). The longitudinal and circular muscle fibers were immunolabeled by Tmod antibodies, displaying a lattice pattern (Fig. 4a). In the viruliferous *N. cincticeps*, Pns10 tubules of RDV extended from or passed through the Tmod-labled microvilli, and a few tubules successfully crossed into the gut lumen at 6-day post-first access to diseased plants (padp) (Fig. 4b). These results were consistent with the previous study revealing that Pns10 tubules associate with and pass through the actin-based microvilli in the midgut of viruliferous leafhoppers^6^. At 12-day padp, abundant Pns10 tubules located Tmod-labled visceral muscles on the outer side (Fig. 4c). Thus, these co-localization studies confirmed that Pns10 tubule interacted with Tmod in cultured leafhopper cells and in intact insects.

### Tmod positively regulates the expression of Pns10

**Figure 5:**
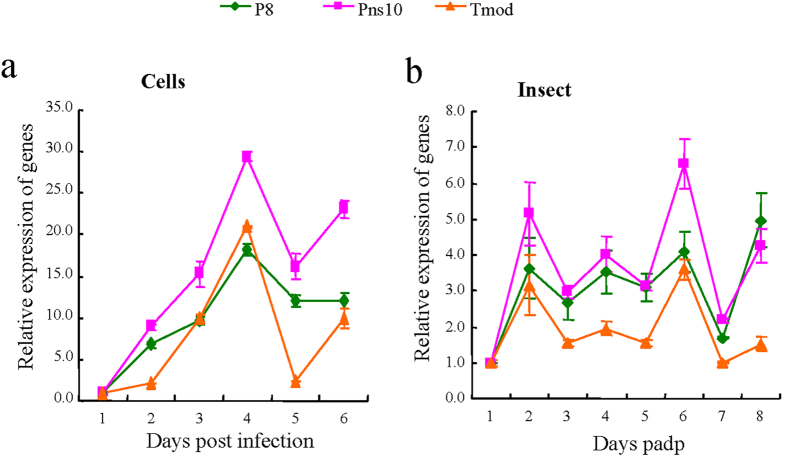
Relative expression profiles of Tmod, Pns10 and outer capsid protein P8 during RDV infection in cultured cells (**a**) and leafhopper (**b**) Cultured cells were inoculated with purified RDV at a MOI of 1 and harvested daily for 6 days. RDV-infected rice plants fed nonviruliferous second-instar nymphs for 3 days, and more than 30 insects were collected daily over a period of 8 days. The results were normalized against the level of the SDHA gene, and the expression levels of Tmod, Pns10 and P8 in RDV-infected cells at 1 dpi and in viruliferous insect at 1-day padp were normalized as 1. Error bars indicate standard deviations from three independent RT-qPCRs.

We then investigated whether the expression of Pns10 of RDV could be mediated by its interaction with Tmod during viral infection in insect vector. The relative mRNA expression of Tmod in RDV-infected cultured cells was analyzed in 6 days post inoculation using RT-qPCR assay, by normalizing its expression levels in RDV-infected cells at 1 day post inoculation (dpi) as 1. RT-qPCR assay demonstrated that the relative expression levels of Tmod, Pns10 and outer capsid protein P8 of RDV were up-regulated at 4 dpi, and then synchronously decreased (Fig. 5a). The expression levels in viruliferous insects also displayed similar trends for Tmod, Pns10 and P8 of RDV in 8 days padp (Fig. 5b). These results indicated that the expression profiles of Tmod tended to be similar to the dynamics of Pns10 expression and RDV accumulation in cultured cells and intact insects during viral infection.

**Figure 6:**
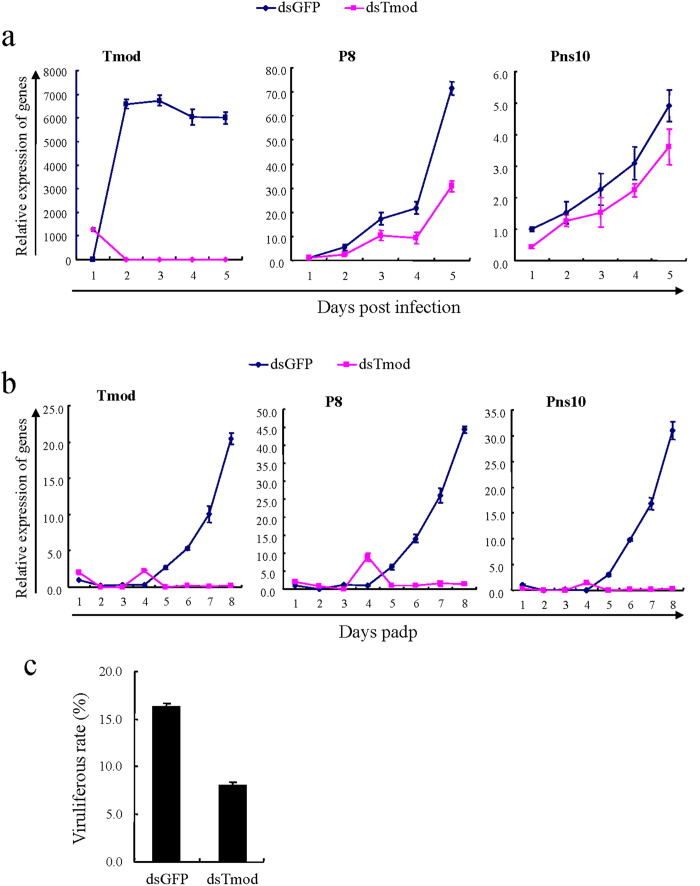
Knockdown of Tmod inhibited Pns10 expression and RDV infection. In cultured cells (**a**) and insect vector (**b**), RNAi induced by dsTmod significantly reduced Pns10 expression and RDV accumulation. At 8 h post transfection with dsGFP or dsTmod, cultured cells were inoculated with RDV and harvested daily for 5 days. Nonviruliferous second-instar nymphs sequentially subjected to dsRNAs microinjection and 3-day acquisition of RDV, then more than 30 alive insect were collected daily for 8 days. (**c**) Knockdown of Tmod decreased the viruliferous rate of *N. cincticeps*. The dsRNA-treated insects which then fed on RDV-infected plant for 3 days were collected for assessing the viruliferous rates by RT-PCR at 14-day padp. The results were normalized against the level of the SDHA gene, and the expression levels of Tmod, Pns10 and P8 in dsGFP-treated cells at 1 dpi and in dsGFP-treated insect at 1-day padp were normalized as 1. Error bars indicate standard deviations from three independent RT-qPCRs.

We then used RNAi experiments to investigate the effect of reduced Tmod expression on viral infection in leafhopper vector. The cultured leafhopper cells were treated with dsRNAs specific for Tmod (dsTmod) and for GFP as control via Cellfectin-based transfection. The viability tests revealed that transfection reagent and dsRNAs caused the absence of toxicity to cultured cells (data not shown). Relative expression of Tmod, Pns10 and P8 of RDV in 5 days post infection was analyzed using RT-qPCR assay. The result showed a significant decrease in the relative Tmod expression in the dsTmod treatment when compared to the dsGFP treatment (Fig. 6a). The relative expressions of P8 and Pns10 in the dsTmod treated cultured cells were also lower than those in the dsGFP treated cells (Fig. 6a), revealing that knockdown of Tmod reduced the expression of Pns10 and accumulation of RDV in cultured cells.

In insect vectors, the effect of dsTmod on Pns10 expression and RDV infection was more significant in 8 days padp (Fig. 6b). The relative expression of Tmod, Pns10 and RDV P8 significantly reduced in dsTmod-treated insects, suggesting that Tmod expression was positively associated with Pns10 expression and viral accumulation (Fig. 6b). Together with the results that Tmod interacted with Pns10 *in vitro* and *in vivo*, it revealed that Tmod specifically regulated Pns10 expression and indirectly affected viral infection. The viruliferous rate of insects was also detected using RT-PCR assay after a circulative transmission period of 14 days. The results demonstrated that knockdown of Tmod significantly reduced the viruliferous rate by more than 50% (Fig. 5c). Our results suggested that the knockdown of Tmod inhibited Pns10 expression and viral accumulation, resulting in the decrease of the viruliferous rate of leafhopper vectors. To summarize, these results suggested that RDV infection up-regulated the Tmod expression, which was beneficial for facilitating viral infection in insect vectors.

## Discussion

The actin filament is an important cytoskeleton protein involved in cellular motility and architecture, and also plays a role in the spread of rice reoviruses in insect vectors^4^,^21^. In the cultured cells of insect vectors, rice reoviruses exploit virus-induced tubules to penetrate into actin-based filopodia, and the ABTM propels the tubules to enter neighboring cells enabling intercellular viral spread^1^,^2^,^3^,^4^,^6^. In the intestines of insect vectors, the ABTM also propels the tubules to overcome membrane or tissue barriers, including microvilli, tight junctions between epithelial cells, and visceral muscles for viral rapid spread^1^,^2^,^3^,^4^,^6^,^16^. In the process of actin-based tubule trafficking, actin-binding proteins such as myosin or cofilin may be recruited by tubule proteins to facilitate viral spread^4^,^16^,^18^,^22^,^23^. However, these interacting proteins have not been further examined in the insect body using molecular techniques, and hence there is lack of direct evidence to support the biological function of these interactors. In this study, putative candidates interacting with RDV Pns10 were screened using YTH assay. The molecular functions of positive colonies with annotated sequences revealed several putative candidates possessing transporter activity, which possibly assisted or guided Pns10 tubule spread (Figs S1 and S2). Based on the high screening frequency and molecular functions of the screened proteins, the following 6 candidates Tmod, Vg, vigilin, LP, Mito P and AIF, were selected for further validation. In the YTH and BiFC assays, positive and strong interactions of Pns10 with insect Vg, LP and Tmod were confirmed (Fig. 1).

Vg is a major yolk protein precursor in insects for the developing oocytes to meet the nutrient requirements during egg development^24^. This female-specific protein is synthesized in the fat body and release into the hemolymph, from where it is absorbed by the growing oocytes^25^,^26^. The transportation of Vg in the circulatory system and endocytosis by the developing oocytes is a rapid and convenient means for viral dissemination and infection. Rice stripe virus (RSV) entry into the oocyte is mediates by direct interaction of Vg with the outer capsid protein of RSV, which is thought to contribute to the transovarial transmission of RSV^27^. RDV is also transovarially transmitted by the leafhopper^4^, however, whether Vg mediated RDV spread or entry into oocytes has not been investigated yet. Our study showed that Vg interacted with Pns10 in YTH system and BiFC assay, suggesting that Pns10 tubules possibly exploited Vg for rapid spread from the fat body to the hemolymph, and also for entry into the oocytes. Transmission electric microscopy and immunofluorescense assays would be required to provide further confirmation.

Lipophorin, a lipid-transport protein, is yet another major yolk protein precursor. Like Vg, lipophorin is synthesized in the fat body, carries lipids in the hemolymph, and is finally taken up by the developing oocytes^26^,^28^. In our study, LP, a precursor of lipophorin, showed 83% identity with the lipophorin precursor of *Nilaparvata lugens*, and interacted with Pns10 in YTH system and BiFC assay. Whether LP carries virus-packaging Pns10 tubules for rapid spread in the hemolymph, or assists virus entry into the oocytes for transovarial transmission also requires further investigation.

**Figure 7:**
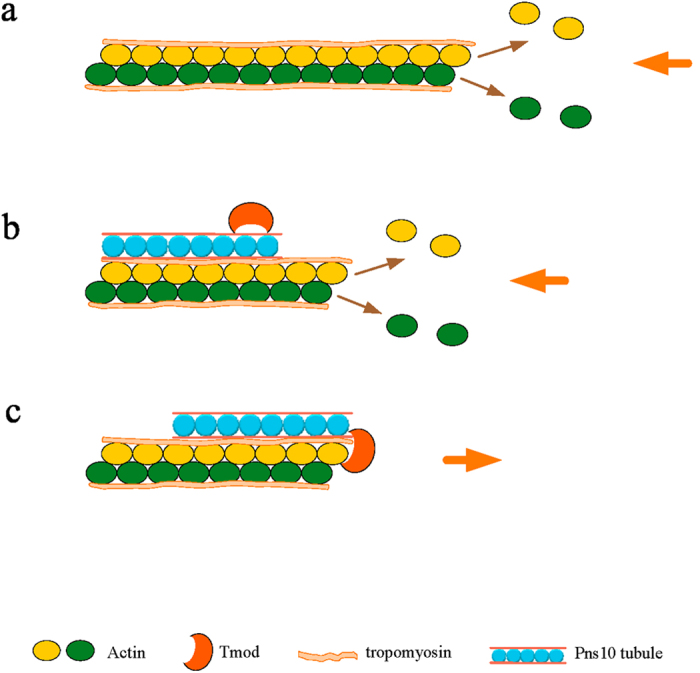
A proposed model for the ABTM protected by Tmod. (**a**) The length of actin-tropomyosin filament was regulated by association and dissociation of actin subunit. (**b**) The Pns10 tubules in infected cells recruited and bond with Tmod which acted as a capping protein to protect the actin filament from dissociation. (**c**) The Tmod exploited by Pns10 tubules blocked the depolymerization at the pointed end, in order to guarantee the growth of actin filament Pns10 tubule trafficking along.

Due to Tmod being an actin-associated protein, we focused on the role of Tmod in the spread of Pns10 tubules of RDV in leafhopper vector. We identified Tmod as a credible interactor with Pns10 both in insect cultured cells and intact insect (Figs 3 and 4). Moreover, Tmod positively regulated Pns10 expression via facilitating the spread of virus-containing tubules (Figs 5 and 6). Tmod is a dynamic capping protein which partly regulates the association and dissociation of the actin subunit, by binding to the pointed end of actin-tropomyosin filament, thus controlling length and stability of the filament^19^,^20^,^29^,^30^,^31^. In previous experiments, we have determined that the elongating of Pns10 tubules depended on the assembly of the actin-tropomyosin filament^2^,^6^,^16^. Based on these invstigations, we proposed a model that Tmod likely protected the actin-tropomyosin filament from depolymerization, and thus facilitated the elongation of Pns10 tubules (Fig. 7). We thus determined that Tmod was involved in the process of ABTM for providing power to propel Pns10 tubules to overcome the membrane and tissues barriers in leafhopper vector (Fig. 7). This interaction may be a conserved strategy evolved by tubules of other plant reoviruses that utilize ABTM for viral efficient spread and transmission by insect vectors.

## Methods

### Cells, viruses and vectors

The non-viruliferous individuals of *N. cincticeps* were collected from Fujian Province, China and propagated for several generations at 25 ± 3 °C in the laboratory. The RDV-infected rice plants were collected from Yunnan Province, China, and propagated via transmission by *N. cincticeps* under greenhouse conditions. The insect cell culture was originally established from embryonic fragments of *N. cincticeps*, and maintained in LBM growth medium for several generations.

### Antibodies

The full-length cDNA of Tmod from *N. cincticeps* was amplified by RT-PCR, and the products were purified and cloned into pHM4 vector. The construct of pHM4-Tmod was then transformed *Escherichia coli* strain BL21 to express proteins His-Tmod induced by isopropyl-β-D-thiogalactopyranoside (IPTG) (Sigma, USA) (1 mmol/L). Cells were harvested and analyzed by SDS-PAGE gel. The protein of expected size isolated from the gel was used for the preparation of mouse polyclonal antisera against the Tmod by the Beijing Protein Innovation Company, which is approved by the Beijing Municipal Science and Technology Commission. IgG was isolated from the specific polyclonal antisera using a protein A-Sepharose affinity column (Thermo Fisher Scientific, USA).

To test the specificity of Tmod antibodies of *N. cincticeps*, total proteins from cultured cells/insects were extracted and separated by SDS-PAGE. Then, the separated proteins were transferred to a polyvinylidene difluoride (PVDF) membrane, and detected with the IgG of Tmod as previously described^32^.

Rabbit polyclonal antisera specific for Pns10 were provided by Dr Toshihiro Omura (National Agricultural Research Center, Japan). IgGs of Tmod and Pns10 were directly conjugated to FITC and rhodamine, respectively, according to manufacturer’s instructions (Thermo Fisher Scientific, USA). The Tmod-FITC and Pns10-rhodamine thus prepared were used for immunofluorescence detection.

### YTH assay

YTH screening was performed using a Matchmake Gold Yeast-two-hybrid system (Clontech, USA) according to the manufacturer’s protocol. A cDNA library of the adults and nymphs of *N. cincticeps* was constructed in the pGADT7 vector as prey plasmid. Full-length cDNA of RDV Pns10 amplified by PCR were cloned in pGBKT7 vector as bait plasmids, which was transformed yeast strain AH109 to confirm the absence of toxic or self-activating. Then the bait and prey were co-transformed the AH109, and transformants were screened on the SD double-dropout (DDO) medium (SD/-Leu/-Trp), SD triple-dropout (TDO) medium (SD/-His/-Leu/-Trp) and SD QDO medium (SD/-Ade/-His/-Leu/-Trp). Positive clones were selected and streaked on QDO/X plates containing X-α-Gal (20 μg/mL) for selection of positive clones. The pGBKT7-53/ pGADT7-T interaction served as a positive control, and the pGBKT7-Lam/pGADT7-T served as a negative control.

### BiFC assays

Gateway® technology (Thermo Fisher Scientific, USA) was applied according to the manufacturers’ protocol to construct expression clones of *N. benthamiana*. The genes of Pns10 and candidate proteins Tmod, Vg, LP, vigilin, Mito P and AIF amplified by PCR were individually cloned into the entry vector pDONR221, then introduced into the destination vectors BiFC vectors YC and YN to generate Pns10-YC, Tmod-YN, Vg-YN, LP-YN, vigilin-YN, Mito P-YN and AIF-YN. Then the plasmids were transformed into *Agrobacterium tumefaciens* strain GV3101, which was infiltrated into leaf tissues of four-week-old *N. benthamiana* plants to perform agrobacterium-mediated transient expression^33^. Plant tissue samples were observed using Leica TCS SP5 inverted confocal microscope as previously described^34^.

### Effects of synthesized dsRNAs on viral infection

DNA fragments spanning about 500–1000 bp segment of Tmod and GFP genes were amplified by PCR using the forward primers and reverse primers which possessed a T7 RNA polymerase promoter with the sequence 5′-ATTCTCTAGAAGCTTAATACGACTCACTATAGGG-3′ at the 5′ terminal. The PCR products were used to synthesize into dsRNAs using the T7 RiboMAX (TM) Express RNAi System (Promega, USA) according to the manufacturers’ protocol. Purified dsRNAs were run on an agarose gel electrophoresis to examine the integrity and quantified using spectroscopy.

Twelve μL cellfectin II Reagent (Thermo Fisher Scientific, USA) and 16 μg dsRNA were respectively diluted in 100 μL LBM growth medium without fetal bovine serum and antibiotics^35^, then gently mixed and incubated at room temperature for 40 minutes for complex formation, as previously described^15^. Thereafter, cultured cells were incubated with the mixture for 8 h, then inoculated with purified RDV at multiplicity of infection (MOI) of 1 at 25 °C for 2 h, and were finally recovered for complete culture.

Nonviruliferous second-instar nymphs were microinjected with dsRNAs (0.5 μg/μL) at the intersegmental region of the thorax, and then fed on RDV-infected rice plants for 3 days, as previously described^15^. The insects were collected on the 1st day of emergence of the adult for RT-qPCR detection, or at 15-day padp for viruliferous rate test.

### RT-qPCR detection

Total RNA was extracted from cultured cells and insects using TRIzol Reagent (Thermo Fisher Scientific, USA) following the manufacturer’s instructions. RT-qPCR primers were designed and tested for efficiency and specificity (Supplementary Table S2). First-strand cDNA was synthesized by forward primers of Tmod with total RNA as template in the reaction mixture containing M-MLV Reverse Transcriptase (Promega, USA). RT-qPCR assays were performed in Mastercycler realplex4 real-time PCR system (Eppendorf) using SYBR Green PCR Master Mix kit (Promega, USA). The succinate dehydrogenase A (SDHA) gene of *N. cincticeps* was used as control for each RT-qPCR assay. Quantitative analyses for relative level of gene expression were analyzed using Microsoft Excel tools.

### Immunofluorescense

The infected cultured cells / intestines of the insects were fixed in 4% paraformaldehyde in PBS, and then permeabalized in 0.2% Triton-X, finally incubated in Tmod-FITC and Pns10-rhodamine, as previously described^14^. The immunolabeled samples were observed using a Leica TCS SP5 inverted confocal microscope.

### GST Pull-down assay

GST Pull-down assay was performed as previously described^36^. Pns10 or P8 gene of RDV was cloned into PGEX-3x to construct a plasmid expressing glutathione S-transferase (GST) fusion protein as bait. These two recombinant proteins were respectively expressed in the *E. coli* stain BL21, of which lysates were then incubated with glutathione-Sepharose beads (Amersham). Subsequently, recombinant protein of His-Tmod was added to the beads and incubated for 2 h, followed by washing the beads with PBS 10 times. Finally, elutes washed from the beads were analyzed with Western blotting assay using GST-tag and His-tag antibodies (Sigma), respectively.

### Co-IP assay

Co-IP assay was performed using the Co-Immunoprecipitation (Co-IP) Kit (Thermo Scientific) according to the manufacturer’s instructions. Purified anti-Tmod antibodies or anti-Pns10 antibodies were added to the resin for immobilization for 2 h at room temperature. Then RDV-infected or mock-infected cultured cells were lysed, and the supernatant was incubated with the antibody-immobilized resin for 1 h at 4 °C. The Co-IP fraction was eluted followed by SDS-PAGE and Western blotting analysis using anti-Pns10 antibodies, or anti-Tmod as primary antibodies and anti-mouse IgG-Alkaline Phosphatase antibody (Sigma) as secondary antibodies.

## Additional Information

**How to cite this article**: Chen, Q. *et al*. Tubules of plant reoviruses exploit tropomodulin to regulate actin-based tubule motility in insect vector. *Sci. Rep.* **7**, 38563; doi: 10.1038/srep38563 (2017).

**Publisher's note:** Springer Nature remains neutral with regard to jurisdictional claims in published maps and institutional affiliations.

## Supplementary Material

Supplementary Information
