# Supplementary material for: Tubules of plant reoviruses exploit tropomodulin to regulate actin-based tubule motility in insect vector
Source: Sci Rep. 2017 Jan 9;7:38563. doi: 10.1038/srep38563 (PMC5220352; doi:10.1038/srep38563)
Supplement: Supplementary Information [file srep38563-s1.pdf]

Tubules of plant reoviruses exploit tropomodulin to regulate actin-based tubule motility in insect vector

Qian Chen[#], Linghua Zhang[#], Yanshuang Zhang, Qianzhuo Mao, Taiyun Wei[*]

Fujian Province Key Laboratory of Plant Virology, Institute of Plant Virology, Fujian Agriculture and Forestry University, Fuzhou, Fujian 350002, PR China

# These authors contributed equally to this work.

*Author to whom all correspondence should be addressed, as follows:

Dr. Taiyun Wei, Institute of Plant Virology, Fujian Agriculture and Forestry University, Fuzhou, Fujian 350002, PR China

Tel: +86-591-83789270. Fax: +86-591-83789439.

E-mail: weitaiyun@fafu.edu.cn

Supplementary Figure Legends

Figure S1. Analysis of sequenced genes of which products interacted with RDV Pns10 in YTH assay. (a) Species distribution percentage of putative interactors. The number of each pie was the percentage of proteins matched from that species. (b) Interaction frequency of 149 putative interactors of *N. cincticeps* from the YTH system.

Figure S2. GO categories of the putative interactors of *N. cincticeps* on molecular function from YTH system. Sixteen functional groups of 47 putative interactors with annotation were identified.

Supplementary Table Legends

Table S1. BLASTX search of Genbank database for putative proteins interacting with Pns10 in the YTH assay.

Table S1. Primers used in this study.

Figure S1

a

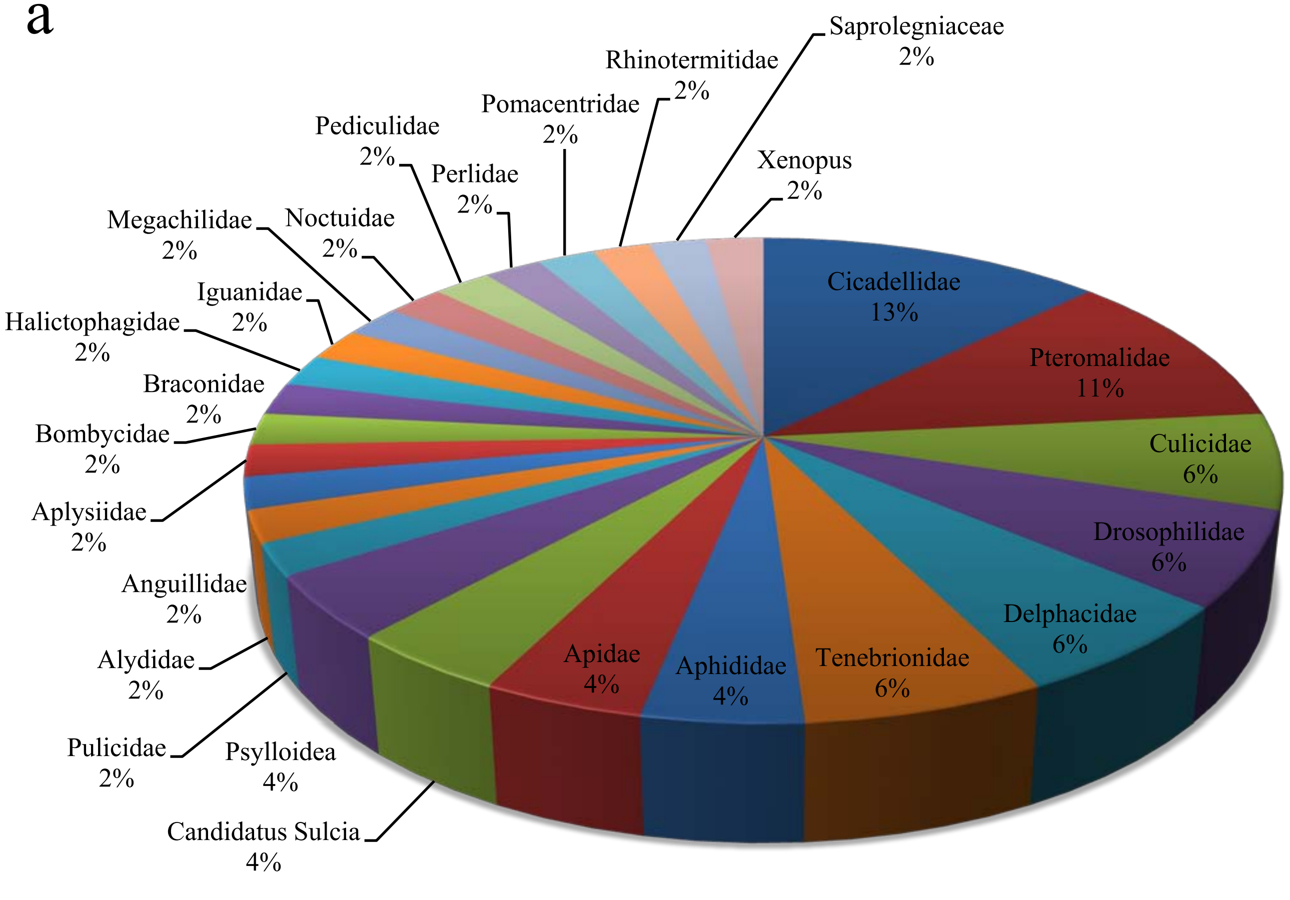
Saprolegniaceae 2%
Rhinotermitidae 2%
Pomacentridae 2%
Xenopus 2%
Pediculidae 2%
Perlidae 2%
Megachilidae 2%
Noctuidae 2%
Iguanidae 2%
Halictophagidae 2%
Braconidae 2%
Bombycidae 2%
Aplysiidae 2%
Anguillidae 2%
Alydidae 2%
Pulicidae 2%
Psylloidea 4%
Candidatus Sulcia 4%
Cicadellidae 13%
Pteromalidae 11%
Culicidae 6%
Drosophilidae 6%
Delphacidae 6%
Tenebrionidae 6%
Aphididae 4%
Apidae 4%

b

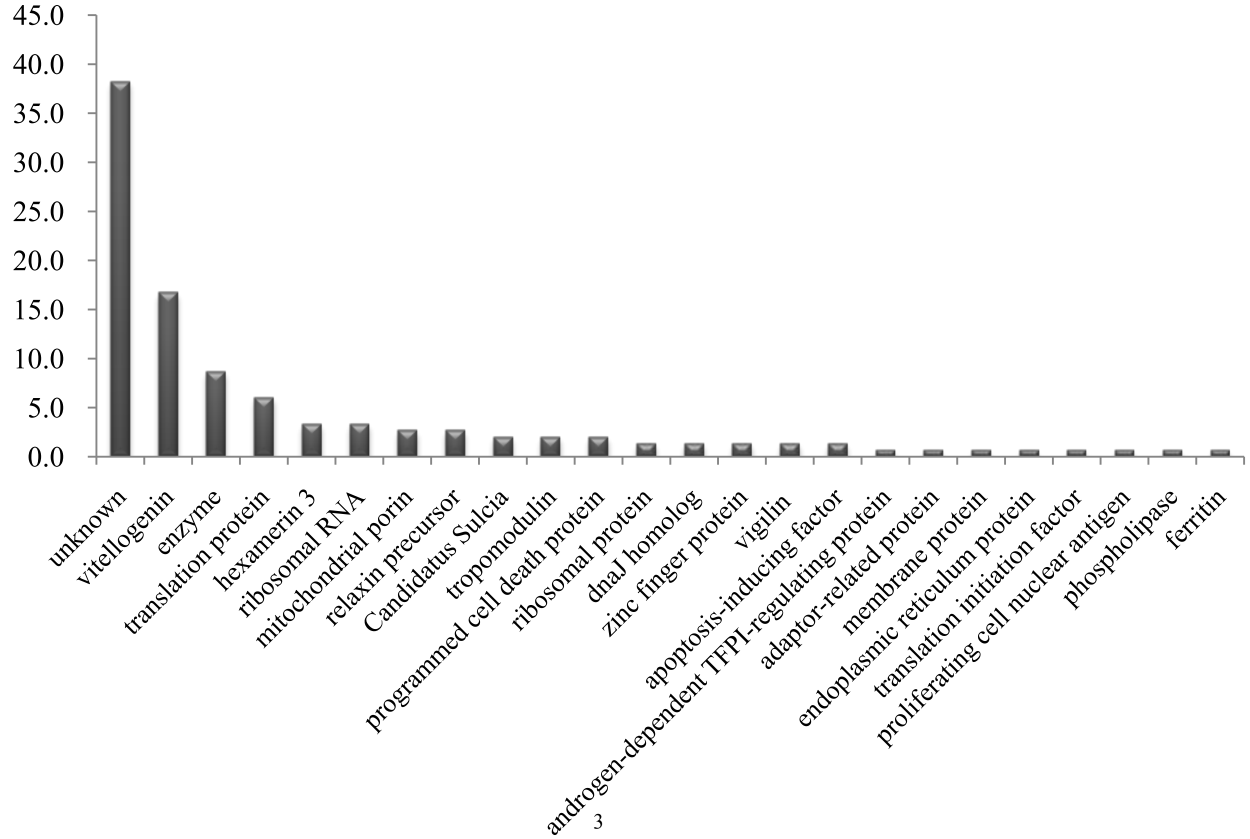
45.0
40.0
35.0
30.0
25.0
20.0
15.0
10.0
5.0
0.0
unknown
vitellogenin
enzyme
translation protein
hexamerin 3
ribosomal RNA
mitochondrial porin
relaxin precursor
Candidatus Sulcia
tropomodulin
programmed cell death protein
ribosomal protein
dnaJ homolog
zinc finger protein
vigilin
apoptosis-inducing factor
androgen-dependent TFPI-regulating protein
adaptor-related protein
membrane protein
endoplasmic reticulum protein
translation initiation factor
proliferating cell nuclear antigen
phospholipase
ferritin

Figure S2

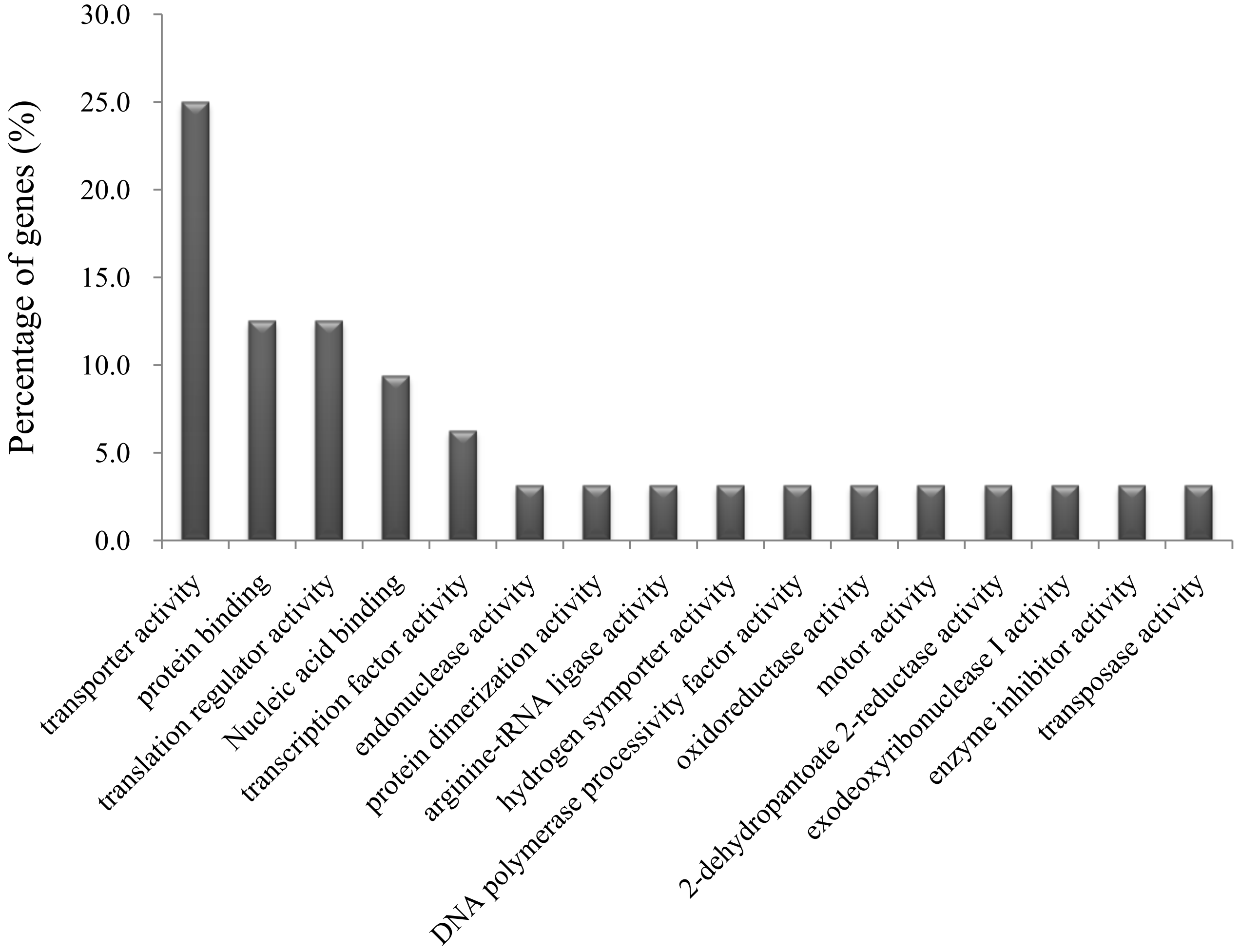

Percentage of genes (%)
30.0
25.0
20.0
15.0
10.0
5.0
0.0
transporter activity
protein binding
translation regulator activity
Nucleic acid binding
transcription factor activity
endonuclease activity
protein dimerization activity
arginine-tRNA ligase activity
hydrogen symporter activity
DNA polymerase processivity factor activity
oxidoreductase activity
motor activity
2-dehydropantoate 2-reductase activity
exodeoxyribonuclease I activity
enzyme inhibitor activity
transposase activity

Table S1. BLASTX search of Genbank database for putative proteins interacting with Pns10 in the YTH assay.

NO.	Accession	Protein	Species	Identities (%)
1	ABM55447	ribosome-associated membrane protein	*Xenopsylla cheopis*	82
2	AM690367	hexamerin 3 (hex3)	*Perla marginata*	81
3	NM_001086581	lysyl-tRNA synthetase (kars)	*Xenopus*	82
4	XM_001659135	high density lipoprotien binding protein / vigilin	*Aedes aegypti*	70
5	EU099536	relaxin 3c precursor (Rln3c)	*Anguilla japonica*	78
6	CU362532	cDNA	*Aphanomyces euteiches*	100
7	XM_001845707	eukaryotic translation initiation factor 3 subunit 6	*Culex quinquefasciatus*	75
8	XM_002021241	GL25245 (Dper\GL25245)	*Drosophila persimilis*	79
9	XM_001360948	uncharacterized protein (Dpse\GA15681)	*Drosophila pseudoobscura*	76
10	XM_001649316	lysyl-tRNA synthetase	*Aedes aegypti*	80
11	AB465596	lipophorin precursor	*Nilaparvata lugens*	74
12	XP_002423791	eukaryotic translation initiation factor 4 gamma	*Pediculus humanus corporis*	80
13	XM_005091162	proteasome subunit beta type-6-like	*Aplysia californica*	75
14	GU123754	28S ribosomal RNA gene	*Nephotettix modulatus*	99
15	CP001981	complete genome	*Candidatus Sulcia muelleri DMIN*	95
16	HQ232817	16S large subunit ribosomal RNA gene	*Halictophagus calcaratus*	77
17	XM_003399740	stress-associated endoplasmic reticulum protein 2-like	*Bombus terrestris*	78
18	XM_003694894	DNA methyltransferase 1 associated protein 1 (DMAP1)	*Nilaparvata lugens*	77
19	XM_003700344	uncharacterized LOC100881888	*Megachile rotundata*	73
20	NM_001162159	bhlhzip transcription factor max/bigmax-like	*Acyrthosiphon pisum*	78
21	L13017	mitochondrion genomic DNA	*Candidatus Sulcia*	91
22	AY588073	vacuolar ATP synthase subunit E	*Homalodisca coagulata*	75
23	KC122909	tropomodulin	*Nilaparvata lugens*	76
24	AK417084	ribosomal protein S6	*Riptortus pedestris*	77

25	KC632288	proliferating cell nuclear antigen	*Coptotermes formosanus*	77
26	XM_004931720	androgen-dependent TFPI-regulating protein-like	*Bombyx mori*	76
27	AY725783	putative ferritin	*Oncometopia nigricans*	84
28	XM_006621927	dnaJ homolog subfamily B member 11-like	*Apis dorsata*	74
29	XM_008120228	apoptosis-inducing factor	*Anolis carolinensis*	86
30	XM_008187441	roadkill (LOC100162530), transcript variant X5	*Acyrthosiphon pisum*	77
31	KF800735	phospholipase C gamma 1 (PLCG1)	*Helicoverpa armigera*	75
32	XM_966236	protein snail	*Tribolium castaneum*	79
33	XM_008194354	programmed cell death protein 8	*Tribolium castaneum*	78
34	XM_008194546	uncharacterized LOC656848 (LOC656848), transcript variant X1	*Tribolium castaneum*	77
35	XM_001605371	zinc finger protein 135-like	*Nasonia vitripennis*	81
36	XM_008206296	J domain-containing protein	*Nasonia vitripennis*	81
37	XM_008218175	adaptor-related protein complex 2, alpha 2 subunit (Ap2a2)	*Nasonia vitripennis*	77
38	XM_008213432	muscle M-line assembly protein	*Nasonia vitripennis*	75
39	XM_008219318	ornithine decarboxylase antizyme 1	*Nasonia vitripennis*	92
40	XM_008302721	RAN binding protein 9 (ranbp9)	*Stegastes partitus*	83
41	XM_008469846	speckle-type POZ protein A (LOC103505505), transcript variant X3	*Diaphorina citri*	80
42	XM_008470604	arrestin domain-containing protein 2 (LOC103506216), transcript variant X1	*Diaphorina citri*	75
43	NM_001298207	transcript variant L (Amun)	*Drosophila melanogaster*	78
44	XM_008546773	uncharacterized LOC103569462	*Microplitis demolitor*	75
45	DQ118408	vitellogenin	*Homalodisca coagulata*	78
46	DQ445519	vacuolar ATP synthase subunit E	*Graphocephala atropunctata*	77
47	DQ445537	mitochondrial porin	*Graphocephala atropunctata*	78

Table S1. Primers used in this study.

Genes	Primers	Sequences (5’-3’)
Tmod	Q-Tmod-forward	CCCGAATGTGATTGTCC
	Q-Tmod-reverse	GTTACCCAGCACCGAAG
	Tmod-forward	attB1 adapter-ATGACGACATCAGCAGCCAAG
	Tmod-reverse	attB2 adapter-CTAGGACGTGGTGGTTTCCTT
	T7-Tmod-forward	T7-ATCCCAAAGGTGTAGTTATG
	T7-Tmod-reverse	T7-CAATCTTGTTACCCAGCA
	pHM4-Tmod-forward	GGTACCATGACGACATCAGCAGCC
	pHM4-Tmod-reverse	CTGCAGCTAGGACGTGGTGGTTTC
vigilin	vigilin-forward	attB1 adapter-ATGCAGCAAGGAGTAGAGGAG
	vigilin-reverse	attB2 adapter-TTAGCGGCGCGGGCCCCATGT
Vg	Vg-forward	attB1 adapter-ATGTCCCTCAACGTATCTGTGG
	Vg-reverse	attB2 adapter-TTATAATAGGTGGATGGCTT
Lp	Lp-forward	attB1 adapter-ATGAAGTTGTACGGTGAATTC
	Lp-reverse	attB2 adapter-TTAGGGATGTTCTGCTGTTAA
Mito P	Mito P-forward	attB1 adapter-ATGGCTCCTCCCACATACGGA
	Mito P-reverse	attB2 adapter-TTAGGCCTCAAGTTCAAGGCT
AIF	AIF-forward	attB1 adapter-ATGTTGAGACCGCTTAGTAAA
	AIF-reverse	attB2 adapter-CTATTCAGAGTGTATGTCAAA
SDHA	Q-SDHA-forward	TGTGGCTGACAGAACAGG
	Q-SDHA-reverse	CAAGTCCAGGGCAAAGTA
RDV P8	Q-P8-forward	TACAGCCATCAGCTAAGCCAAA
	Q-P8- reverse	CCGCAACAGACCGAAACA
GFP	T7-GFP-forward	T7-ATGTGCTGCAACATGAGCTCAC
	T7-GFP- reverse	T7-TTACGCAAAGTACATGACTTTCTTG
	PGEX-GFP-forward	CCCGGGATGAGTAAAGGAGAAGAACT
	PGEX-GFP-reverse	GAATTC TTATTTGTATAGTTCATCCATG
Pns10	PGEX-Pns10-forward	CCCGGGATGGAAGTAGACACTGCTAC
	PGEX-Pns10-reverse	GAATTCTTAGGAACCGCCGCCTTTAA

T7 sequence: ATTCTCTAGAAGCTTAATACGACTCACTATAGGG.
Prefix T7 indicates primers used in RNA silencing.
Prefix Q indicates primers used in RT-qPCR
attB1 adapter sequence: GGGGACAAGTTTGTACAAAAAAGCAGGCTTC
attB2 adapter sequence: GGGGACCACTTTGTACAAGAAAGCTGGGTC

Figure S3

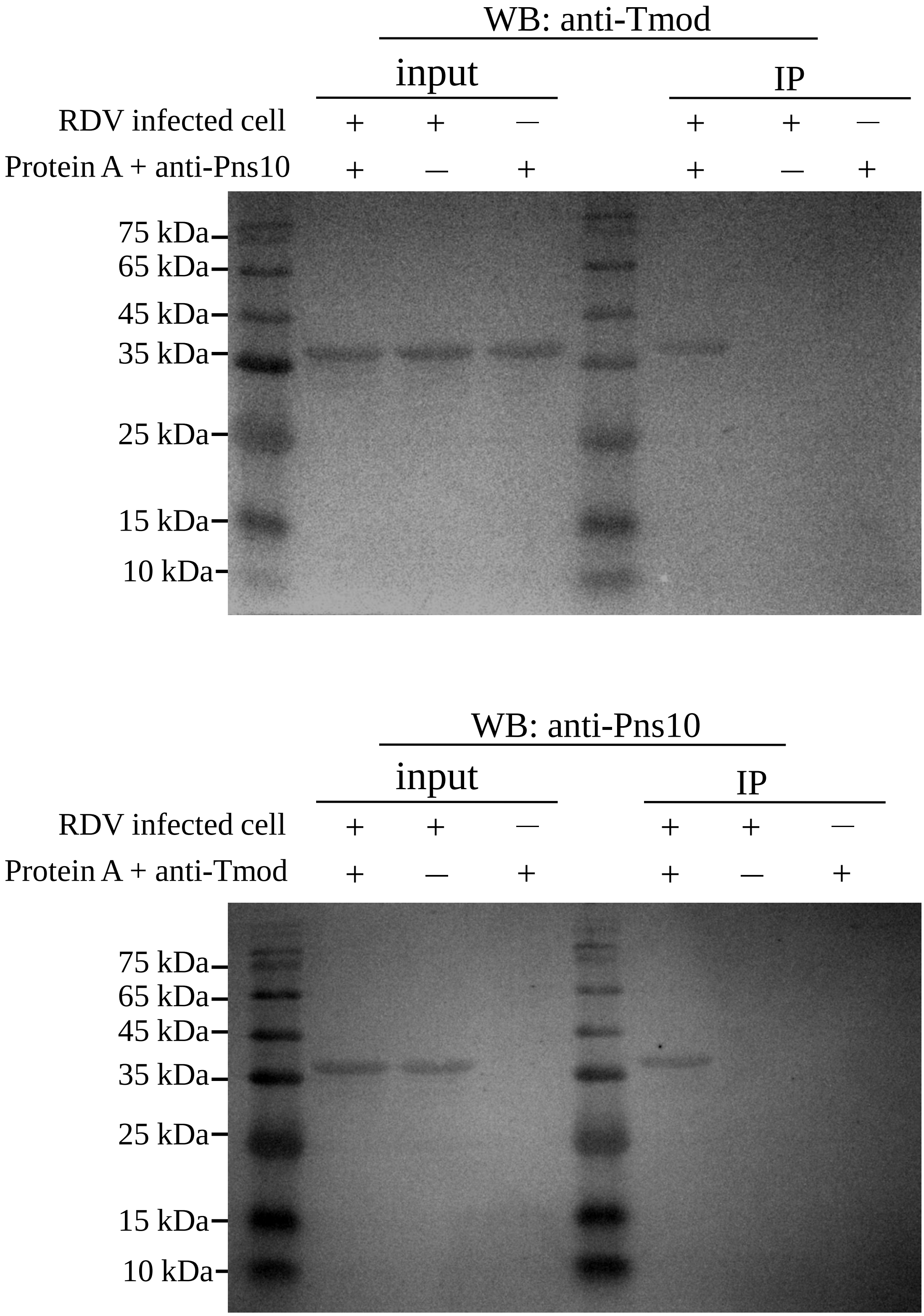
WB: anti-Tmod
input
IP
RDV infected cell + + — + + —
Protein A + anti-Pns10 + — + + — +
75 kDa
65 kDa
45 kDa
35 kDa
25 kDa
15 kDa
10 kDa
WB: anti-Pns10
input
IP
RDV infected cell + + — + + —
Protein A + anti-Tmod + — + + — +
75 kDa
65 kDa
45 kDa
35 kDa
25 kDa
15 kDa
10 kDa
